# Setting up crowd science projects

**DOI:** 10.1177/0963662516678514

**Published:** 2016-11-30

**Authors:** Kaja Scheliga, Sascha Friesike, Cornelius Puschmann, Benedikt Fecher

**Affiliations:** Alexander von Humboldt Institute for Internet and Society, Germany; Hans Bredow Institute for Media Research; German Institute for Economic Research & Alexander von Humboldt Institute for Internet and Society, Germany

**Keywords:** case study research, citizen science, crowd science, crowdsourcing, digitisation of research, online platforms, science communication, volunteer engagement

## Abstract

Crowd science is scientific research that is conducted with the participation of volunteers who are not professional scientists. Thanks to the Internet and online platforms, project initiators can draw on a potentially large number of volunteers. This crowd can be involved to support data-rich or labour-intensive projects that would otherwise be unfeasible. So far, research on crowd science has mainly focused on analysing individual crowd science projects. In our research, we focus on the perspective of project initiators and explore how crowd science projects are set up. Based on multiple case study research, we discuss the objectives of crowd science projects and the strategies of their initiators for accessing volunteers. We also categorise the tasks allocated to volunteers and reflect on the issue of quality assurance as well as feedback mechanisms. With this article, we contribute to a better understanding of how crowd science projects are set up and how volunteers can contribute to science. We suggest that our findings are of practical relevance for initiators of crowd science projects, for science communication as well as for informed science policy making.

## 1. Introduction

Online technologies help to organise thousands of people around the world who engage in the process of knowledge creation (Castells, 2000; Dickel and Franzen, 2016; Meyer and Schroeder, 2015; Stehr, 2002). This enriches institutionalised science and offers new ways of engaging with civil society (Bonney et al., 2016; Riesch and Potter, 2014).

Volunteer engagement in knowledge creation is by no means a new phenomenon, but it is one that has gained momentum with the emergence of online technologies. In recent years, a multitude of projects have been launched that allow for interaction between volunteers and professional scientists. As it is analogous to crowdsourcing in applied research, this form of interaction has been labelled ‘crowd science’ (Franzoni and Sauermann, 2014).

What older forms of citizen science and today’s crowd science have in common is that the volunteers’ participation is self-selected and serves the purpose of discovery in both cases (Haklay, 2013; Reed et al., 2013; Silvertown, 2009). But there are some features that differentiate crowd science from traditional citizen science: it is scalable, volunteers participate independently of time and place and the forms of participation involved are novel, such as gamification. Scientific problems can be efficiently distributed among a large number of volunteers who contribute to the discovery process through particular research-related tasks, ranging from data gathering to the interpretation and annotation of datasets.

Modern crowd science is sometimes described as a method of scientific discovery that academic scientists can employ to solve complex research questions (Nature, 2015). It can also be understood as a form of communication and knowledge exchange between volunteers and scientists (Bonney et al., 2016). In this sense, crowd science is neither merely a method of academic research to solve increasingly complex problems nor solely the work of enthusiasts outside of academia. Crowd science appears to complement rather than supplant the role of the professional scientist (Dickel and Franzen, 2016).

Given the potential of crowd science in an academic context, it is hardly surprising that the phenomenon itself has attracted the attention of academic research (Franzoni and Sauermann, 2014; Wechsler, 2014). While scholarly interest in the phenomenon mainly focuses on the organisational principles of crowdsourced research and user motivation, we know little about how these projects come about, and there is limited research critically assessing the implicit understandings held by those running crowd science projects (Dickel and Franzen, 2016). Even though some claim that crowd science is a method of academic discovery (Nature, 2015; Nielsen, 2012), to our knowledge, no research has examined how crowd science projects are set up and what strategies are used to interact with the crowd.

On the basis of case studies of 12 German crowd science projects, we show and reflect upon the different objectives of crowd science projects. We also identify strategies for accessing the crowd, types of participation, approaches to quality assurance and feedback mechanisms. With this article, we hope to shed light on how crowd science projects are set up and on the relationship between the volunteers and professional scientists. In the following sections of this article, we categorise the relevant literature into four perspectives on crowd science; we then describe our methodological approach, present our results and subsequently discuss them.

## 2. Background

### Citizen science and crowd science

Citizen science is generally understood as scientific research that actively involves amateurs or non-professionals in science. There is a long history of non-professional involvement in some domains of research, in some cases predating the professional scientific organisational structures that support modern science. Different philosophies of citizen science persist, depending on national and historical contexts. Along these lines, citizen science is associated with Irwin’s (1995) call to democratise science and draw public attention to issues such as sustainability. Yet, citizen science is also associated with a more practical perspective, assumed, for example, by Bonney (1996), who sought out volunteers to support data collection efforts in ornithology. Both approaches seek to benefit volunteer communities and professional scientists alike. For volunteers, these benefits may include gains in specific knowledge about science and an increased awareness of vital scientific and societal issues. This form of participation may also make the general public more effective in shaping both science and science policy (Bonney et al., 2016; Land-Zandstra et al., 2016). Professional scientists may gain analogous benefits, such as a deeper understanding of how the public interprets scientific problems and what motivates scientific hobbyists, as well as practical benefits, for instance, help with coding large volumes of measurement data (Riesch and Potter, 2014).

Crowdsourcing – the concept of obtaining services, ideas or content through the contribution of a large group of people, typically from online communities (Merriam-Webster, 2016) – rose to prominence in the early 2000s (Howe, 2006). Since then, the concept has been applied to a range of fields, from disaster relief (Zook et al., 2010) to visualisation design (Heer and Bostock, 2010). Academia has intensively studied crowdsourcing as a tool to solve creative problems (Afuah and Tucci, 2012; Bayus, 2013; Leimeister et al., 2009; Poetz and Schreier, 2012) by drawing on the diversity of perspectives inherent in a crowd.

Looking at both citizen science and crowdsourcing, Franzoni and Sauermann (2014) defined *crowd science* as research that is characterised by two features: ‘participation in a project is open to a wide base of potential contributors’ and ‘intermediate inputs such as data or problem solving algorithms are made openly available’ (p. 1). Applied industry research and basic academic research generally differ in their aims, and accordingly, the nature of crowdsourcing and crowd science differs, too. In contrast to crowdsourcing, where payments and prizes are common, crowd science projects heavily depend on volunteers who do not receive any form of pay. Furthermore, many crowd science projects incorporate volunteers in the collection or annotation of scientific data, although some crowd science projects are also concerned with more educational objectives (Bonney et al., 2016). Given these observations, crowd science is not discussed as a specific form of crowdsourcing, but rather as a specific form of citizen science.

That being said, crowd science can be both a way of enhancing citizen participation in science through technology and a mechanism for exploiting the crowd to perform simple and repetitive tasks. The latter resonates with more recent conceptions of the digitisation of science (science 2.0) as a neo-liberal project (Mirowski, 2011) in which discovery is ruled by the primacy of efficiency and where knowledge is not considered a public good but a commodity to be monetised. Following this understanding, crowd science would be a tool to exploit resources for non-public gains. In contrast, it is worth noting that crowd science can also be a means to conduct research projects that would stand little chance of securing funding from conventional research funds or from commercial investors.

To summarise, crowd science can be an effective tool for scholarly communication and an example of good scientific practice; it can, however, also be a euphemism for crowd exploitation.

### Research on crowd science

In our survey of existing literature, we identified four streams of research on crowd science. *The first stream* of research involves case studies and analyses of individual crowd science projects. *The second stream* of research takes a utilitarian view that loosely corresponds to Bonney’s (1996) hands-on approach and considers potential fields of application for crowd science. *The third stream* of crowd science literature critically examines the concept against the background of the ‘problem of extension’ (Collins and Evans, 2002; Dickel and Franzen, 2016), that is, the notion that in order for science to retain a degree of exclusivity and epistemic superiority over other forms of knowledge production, there needs to be limits on who can participate. Finally, *the fourth stream* of research looks at crowd science from the perspective of its democratic potential to enable scientific citizenship in Irwin’s (1995) sense.

### The individual project perspectives on crowd science

The first stream of research focuses on individual crowd science projects. Brossard et al. (2005) analyse the impact of the crowd science project *The Birdhouse* through a user survey. The authors show that participation in the crowd science project increased the participants’ knowledge of bird biology. The authors note that the primary motivation for participants to take part in the project was an interest in birds rather than a desire to contribute to a scientific project. Sullivan et al. (2009) present the bird observation network *eBird*. They describe the community of birdwatchers as ‘a global network of avian sensors’ (p. 2290) and make the observation that bird users have shifted away from making simple causal observations and have begun to provide useful effort-based data. This, according to the authors, shows that long-standing volunteer efforts can be productively combined with new initiatives and technologies. Moreover, the ability to create, manage and manipulate vast, real-time data resources influences the ways in which people study biology and conduct observational research. Raddick et al. (2010) analyse user motivation in the crowd science project *Galaxy Zoo*, an astronomy project that invites people to assist in the classification of large numbers of galaxies. While the authors note that most users’ motivation is multifaceted, the most common motivation was an intrinsic interest in the topic of astronomy, echoing the results of Brossard et al. (2005). Delaney et al. (2008) use quantitative measures of accuracy on the data collected by volunteers. They see the integration of volunteers as a way of establishing large datasets, but they also point to the issue of maintaining the motivation to participate longitudinally, a point that is also raised by Riesch and Potter (2014) in an interview study that examines the views of professional scientists on citizen science. Furthermore, Delaney et al. (2008) highlight funding issues and data quality as relevant problems of crowd science projects.

To summarise, single case studies address a range of questions relating to particular aspects of individual projects. The topics covered include the transformation of citizen science into crowd science through digital infrastructures, the learning experiences of crowd scientists and the motivational aspects of participants. By and large, however, the cited studies do not allow us to draw inferences regarding the impact of crowd science beyond a single platform and do not establish commonalities between different initiatives.

### Utilitarian perspectives on crowd science

The second stream of research assumes a utilitarian stance on crowd science. From this point of view, crowd science is either a means of facilitating data-intensive research or fostering science communication. Hochachka et al. (2012) describe crowd science as a means of promoting data-intensive research in ecology. They see volunteer engagement as a way to extend methods for acquiring, integrating and modelling massive quantities of diverse data. In the same vein, Dickinson et al. (2010) see crowd science as a means to pursue new, quantitative approaches to emerging questions about the distribution and abundance of organisms across space and time. In their opinion, crowd science is a research tool with the capacity to contribute to long-term data-intensive research projects. The authors highlight the requirement for expertise in working with large, messy datasets and the need to have competing hypotheses to make good use of volunteers in research. Cooper et al. (2007: para 1) describe crowd science as a tool for conservation in residential ecosystems. They propose an extension of crowd science that increases the collaboration between researchers and the public: ‘By involving citizen participants directly in monitoring and active management of residential lands, citizen science can generate powerful matrix management efforts, defying the “tyranny of small decisions” and leading to positive, cumulative, and measurable impacts on biodiversity’.

To summarise, the utilitarian perspectives regard crowd science as a method for collecting data or as a tool for science communication. Implicitly, these perspectives assume that while crowd science produces tangible results, it has little impact on professional scientists and their authoritative role in relation to non-professionals.

### Critical perspectives on crowd science

The third stream of literature focuses on critical perspectives on crowd science. Dickel and Franzen (2016) argue that crowd science represents an instance of the ‘problem of extension’ described by Collins and Evans (2002) as the implicit understanding that science’s epistemic dominance over other forms of knowledge depends on its exclusivity, that is, the expertise of professional scientists, which does not fully extend to interested volunteers, who may very well support scientific research, but cannot replace professionals. In a recent book, Collins critically asks whether ‘we [are] all scientific experts now’, to which he answers with a resounding ‘No’ (Collins, 2014). Following this line of thought, volunteer participation in crowd science does not democratise science. It gives rise to alternative forms of knowledge creation that are not in competition with established professional science but that do not contribute to it either. Alternatively, it represents a form of research outsourcing through which the utilitarian potential of volunteer labour is harnessed by professional scientists, a mechanism which may have adverse effects on institutional science in the long term. Dickel and Franzen (2016) accordingly speak critically of a ‘dissolution of social boundaries in the societal knowledge production, which pushes into the background the institutional framework of scientific organizations, professional communities and the professional role of scientists itself’ (p. 11).

In summary, in the literature that delineates critical perspectives on crowd science, the phenomenon is described as the ‘problem of extension’. Blurring the boundaries between crowd science and institutional science, however, might ignite critical reflection as to the strengths and weaknesses of both knowledge creation frameworks.

### Democratising perspectives on crowd science

Finally, the fourth stream of literature looks at crowd science from the perspective of its democratic potential. Franzoni and Sauermann (2014) present examples of crowd science from different fields of science and illustrate similarities regarding what these projects do and how they are organised. They distinguish crowd science from traditional (Mertonian) science by virtue of its open research process, in which intermediate results are shared with a community, which includes non-professionals. In a follow-up study, Sauermann and Franzoni (2015) analyse data from seven different crowd science projects regarding user contribution. They find that most contributors participate only briefly and with little effort, but a small share of users returns on a regular basis to contribute the lion’s share of the work. According to the authors, top contributors also tend to work more efficiently, which suggests a level of learning rather than inherent skill. Tulloch et al. (2013) use return-on-investment thinking to identify the minimum investment needed for different crowd science programmes and the point at which investing more in crowd science has diminishing returns. According to the authors, longitudinal schemes are more cost-effective. Silvertown (2009) argues that the major difference between modern crowd science and the historical form of citizen science is that crowd science is now an activity that is potentially available to all citizens and not just the privileged few. Conrad and Hilchey (2011) review the literature on crowd science and identify two major research gaps. The first gap refers to the need to compare and contrast the success of crowd science projects, a point also made by Bonney et al. (2016). The second gap refers to the need for more research on the use of data from crowd science projects by decision-makers.

While there is already a notable body of research on the motivational aspects of crowd science, its organisational principles and practical fields of application, there is little empirical research on the managerial aspects of crowd science. It is important to understand the views, motivations and strategies of project coordinators, especially against the backdrop that crowd science can be a euphemism for using citizens as free labour. This makes it all the more necessary to address the project management side of the phenomenon and deepen our understanding of how these projects are set up.

## 3. Methodology

Crowd science is a contemporary phenomenon that has received little research attention with regard to its managerial structure. Thus, we chose an exploratory approach for our research design. We conducted a multiple-case, comparative and inductive study (Yin, 2013) as this method was the most appropriate one for achieving our general goal of developing theoretical explanations for why and how crowd science projects emerge.

### Sample

For our research, we limited the case selection to projects from Germany for several reasons. First, Germany has a long tradition of citizen science (Finke, 2014). Second, many examples of crowd science come from English-speaking countries, leaving it unclear whether their findings can be generalised to other contexts. Third, our knowledge of the national research environment allowed us to meet with key people from each case and to conduct face-to-face interviews.

In a first step, we identified 29 research projects that fit the description of crowd science provided by Franzoni and Sauermann (2014). For each project, we collected general information concerning the scope of the project, the responsible entities, the tasks the crowd conducts, the location for the tasks, the target audience and the form of prior knowledge necessary to participate.

Our case selection was led by our goal of developing an understanding of how crowd science projects emerge (Eisenhardt and Graebner, 2007). It was therefore necessary to select individual cases that provided unique insights while contributing to a more general understanding of crowd science. Given the variety of research settings and forms of participation in crowd science projects, we conducted in-depth case studies of 12 crowd science projects from the larger sample. We conducted the case studies iteratively and step-wise. New cases were added to rule out alternative explanations (Eisenhardt and Graebner, 2007). We added cases to our sample as long as they could justify a theoretical contribution. Table 1 provides a summary of the cases we included in our sample.

**Table 1.** Background.

Project	Website	Organisation	Aim	Objective	Open Participation	Open Access
Animal Tracker (AT)	http://www.orn.mpg.de/animaltracker	*Max-Planck-Institut für Ornithologie, Vogelwarte Radolfzell*	Tracks the routes of wild animals, especially birds; animals are tagged with GPS transmitters Data are collected in database (Movebank) Volunteers can track movement of animals via app	Research objective: collecting data on animal movements and insights about animal behaviour Secondary objective: involving the public in research via outreach activities Driven by research question	Anyone can participate	Data contributed by volunteers are published in a database (Movebank) which is publicly accessible Infrastructure is available to all users and researchers
ARTigo – *Laien beschreiben Kunstwerke* (AG)	http://www.artigo.org/	*Institut für Kunstgeschichte der Universität München, Institut für Informatik der LMU*	Improving image search of paintings through tagging Extracting implicit knowledge from tags Creating a platform for similar projects	Research objective: Data analysis Insights for computer science: games design, machine learning Insights for digital humanities: behavioural and cognitive differences observable through annotation of paintings Data-driven approach without specific research question at the outset	Anyone can participate	Data contributed by volunteers are immediately integrated into image search
*Bildungsexplosion* (BE)	http://www.bildungsexplosion.de/	*Bildungsexplosion*	Establishing a knowledge portal with short entries on educational topics The relation between topics is shown by means of interactive graphics	Educational objective: providing short entries for learning about specific topics Interest-driven; no research question	Anyone can participate (but not many people do)	Entries contributed by volunteers are checked in editorial process and, if approved, uploaded onto project website Website is freely accessible
DES – *Datenerfassungssystem* (DES)	des.genealogy.net	*Verein für Computergenealogie e.V.*	Digitising of historical sources (e.g. lists of war casualties) and archiving them in a database	Interest objective: Insights are relevant for historically interested people and have an application for historical research Interest-driven; no research question	No requirements, but volunteers have to learn to read the writing, therefore varying levels of task complexity	Data contributed by volunteers is published in a database and can be accessed
GOV – *Genealogisches Ortsverzeichnis* (GOV)	gov.genealogy.net	*Verein für Computergenealogie e.V.*	Building a database consisting of an index of places relevant for genealogy	Interest objective: Insights are relevant for historically interested people and have an application for historical research Interest-driven; no research question	No requirements, but knowledge in the subject matter is preferable for more complex tasks	Results are published under creative commons Data contributed by volunteers are published in a database and can be accessed
KLEKs (*Das Kulturlandschafts-Wiki*) (KW)	https://www.kleks-online.de/	*Institut für Kulturlandschaftsforschung e.V.*	Digitising cultural landscape elements and archiving them in a database	Interest objective: preservation of information about cultural landscape elements Insights are relevant for people interested in history and cultural landscapes with applications for environmental policy decisions, landscaping, tourism No research question; interest-driven	Anyone can participate Interest in the field is desirable because it helps to avoid erroneous submissions	Entries contributed by volunteers are published in a database and can be accessed Registration is required for certain data Data should not be used for commercial purposes
*Meilensteine der motorischen Entwicklung im Kleinkindalter* (ME)	http://www.ifp.bayern.de/projekte/monitoring/meilensteine.php	*Staatsinstitut für Frühpädagogik München* (IFP)	Collecting empirical data for a study on the motor development of babies	Research objective: establishing empirical norms in child growth Driven by research question	Only parents with babies (up to 12 months) can participate	Data are anonymised and published in scientific and popular scientific outlets
*Mückenatlas* (MA)	http://www.mueckenatlas.de/	*Leibniz-Zentrum für Agrarlandschaftsforschung ZALF; Friedrich-Loeffler-Institut, Bundesforschungsinstitut für Tiergesundheit FLI*	Monitoring mosquitoes and transmittable diseases, and building a reference collection	Research objective: tracking the development of mosquito populations and transmittable diseases, and building a reference collection Driven by research question	Anyone can participate	Pointer to where the sample was collected is added to the website Data will be publicly available after the project results are published
*Open Philology* (OP)	http://www.dh.uni-leipzig.de/wo/open-philology-project/	*Digital Humanities, Universität Leipzig*	Translating ancient Greek and Latin texts	Education objective: possibility of integration into language courses Science objective: enhancing the capabilities of natural language processing (NLP); creating a method that is applicable to all ancient languages Driven by research question	Theoretically anyone can participate, however there is a certain skill level required; volunteers have to take an online Greek or Latin-course first Online language course is supposed to enable everyone to contribute; the tasks vary in difficulty, starting with simple words and ending with grammatically complex texts	Open data access is the goal, but it is not clear how it can be achieved yet
*Tauchen für den Naturschutz* (TN)	http://www.nabu-naturschutztauchen.de/projekt/	*NABU Gransee e.V.*	Monitoring water quality of lakes	Interest objective: monitoring water quality in lakes which is relevant for both recreational divers and environmentalists Project brings both groups together who have often conflicting interests No research question; interest-driven but data can be used for scientific and environmental purposes	Anyone can participate (diving skills required)	Data contributed are added to database and can be accessed
*Verlust der Nacht – App* (VN)	http://www.verlustdernacht.de/	*Leibniz-Institut für Gewässerökologie und Binnenfischerei* (connected with citizen science project GLOBE at Night)	Measuring sky glow in order to examine implications of light pollution	Research objective: measuring sky glow around the world to assess the changes over time Driven by research question	Anyone can participate (smart phone needed)	Data are anonymised and uploaded onto *GLOBE at Night* database Platform operator is writing a guide explaining how to use the data
*WISSENSDINGE. Geschichten aus dem Naturkundemuseum Berlin* (WD)	http://www.mfn-wissensdinge.de/	*PAN – Perspektiven auf Natur, Museum für Naturkunde Berlin*	Documenting stories about objects from the Natural History Museum in Berlin	Research objective: showing different points of view on objects, educational aspect Engagement with museum content No research question	Anyone can participate	Stories are edited and made available on project website Most interesting stories are published in a book

### Data collection

Our main data source was semi-structured interviews with project managers and other involved individuals. We used further data sources such as additional short and informal interviews, observational data, workshop and conference impressions, journalistic reports, publications and archival data from the projects (e.g. blog posts, press releases) for data triangulation (Eisenhardt, 1989). This form of triangulation allowed us to enhance construct validity by cross-checking facts and questioning isolated statements (Jick, 1979).

During our in-depth interviews with representatives of the selected cases, we asked respondents about past and current events to obtain an understanding of how the project evolved. We organised all interviews according to a semi-structured interview guide and gave all interviewees space to add further information. Questions were focused on facts and events rather than interpretations and opinions. To control for respondent bias, we did not present our emergent theoretical model to the interviewees.

All interviews were conducted during the second half of 2014 in the German language; they lasted between 30 and 65 minutes and were recorded. Every interview was then transcribed verbatim, which led to an interview report of approximately 65,000 words.

We asked our interview partners about the project’s background, which included a general description of the project and the rationale behind it, and enquired about the role the Internet plays for the project. Next, we focused on questions concerning the volunteers and asked the interviewees to describe these individuals and to tell us about the strategies employed to involve them and to keep them engaged. We also asked about barriers to entry and the handling of data generated by the volunteers. Finally, we enquired about quality control and feedback mechanisms.

### Data analysis

We entered the collected data into a case database and developed individual case reports. These reports included information from all sources, individual quotes, observations, timelines and key facts. On the basis of the interviews and our case reports, we conducted within-case analyses. We thematically coded the available information and formed independent views for each case history. In a series of workshops, conducted at regular intervals throughout 2015, we discussed these views and cross-checked our emerging theoretical contribution. This provided us with the opportunity to verify facts and work out inconsistencies in our theoretical understanding. Our cross-case analysis was conducted by entering data from all case reports into thematically structured tables which helped us to conduct cross-case pattern sequencing (Miles and Huberman, 1984) and pair-wise comparisons (Eisenhardt, 1989).

Through our research, we identified several core challenges that crowd science platforms have to deal with in their development phase. Furthermore, we wanted to describe how these challenges can lead to different decisions and therefore to differences among crowd science platforms.

## 4. Results

Our data analysis revealed five aspects that are relevant for setting up a crowd science project. *First*, we delineate the objectives of crowd science projects, differentiating between those where the main objective is knowledge generation and those where the main objective is centred on an interest in a topic. Furthermore, within projects where the primary objective is knowledge generation, we distinguish between approaches that are driven by a research question and projects that pursue a data-driven approach. *Second*, we describe ways of accessing the crowd. *Third*, we classify the tasks performed by the volunteers into three broad categories: annotating, collecting and producing. *Next*, we discuss the issue of quality assurance and differentiate between manual and automated mechanisms. *Finally*, we discuss feedback mechanisms. We present an overview of our findings in Table 2 and describe our findings in more detail in the following sections.

**Table 2.** Results.

Objective			Crowd access		Task			Quality assurance		Feedback mechanism	
Knowledge generation		General interest	Building	Harnessing	Annotating	Collecting	Producing	Manual	Automated	Individual	Conversational
Question driven	Data driven										
AT	AG	BE	AG	AT	AG	AT	WD	TN	AG	BE	(AT)
ME	GOV	TN	BE	TN	DES	ME	BE	MA	OP	ME	(AG)
MA	DES	KW	DES	OP	GOV	MA	OP	WD	VN	MA	DES
OP	VN	WD	GOV		DES	TN		BE		TN	GOV
			KW			KW		DES			KW
			ME					AT			OP
			MA					GOV			VN
			WD					KW			
			VN					ME			

The notion of *open participation* and *open access* to the project’s results, as described by Franzoni and Sauermann (2014), is reflected in the crowd science projects we studied. In most projects we analysed, there are no formal restrictions in terms of participation (for exceptions, see Table 1). Furthermore, most projects share intermediate inputs by immediately adding volunteer contributions to their database and most projects make the data openly accessible. An overview of the projects’ level of open participation and open access to results can be found in Table 1.

### The objectives of crowd science projects

All crowd science projects engage volunteers to perform specific tasks, typically generating or processing some form of data that contributes to the overall goal of the project. Yet, there are differences regarding the exact aims and objectives. We distinguish between two project forms: projects where the predominant objective is the *generation of knowledge* and projects where the *general interest in a topic* is the key concern. Among projects where the main objective is to generate knowledge, we differentiate further between projects that are initiated by scientists who want to employ crowd science as a means of answering a specific research question (e.g. *Mückenatlas*) or by scientists who have a research interest in a specific area and pursue a data-driven approach without a specific research question at the outset (e.g. *ARTigo*). Crowd science projects where a general interest is the main objective are typically initiated by an individual or a group of people who are passionate about a topic and who want to share their enthusiasm with others. As one project coordinator explained,

But what is in my opinion, very, very important and also distinguishes the project is that it did not come from a scientific institute. […] In our case the citizens themselves developed the project without cooperating with an academic institution. (*Tauchen für den Naturschutz*)

While these projects are also centrally concerned with knowledge generation, they focus on making materials and data on the topic of interest available to others. For example, the project *Tauchen für den Naturschutz* gathers data that are useful for monitoring the water quality of lakes. This is of interest to the communities involved, such as scuba divers and environmentalists. Other cases in our sample that centre on a topic of interest are *Bildungsexplosion, KLEKs* and *WISSENSDINGE*.

Regardless of whether project initiators are driven by the desire to include volunteers to help them generate knowledge or by a desire to engage volunteers in a topic of shared interest, all crowd science projects rely on the engagement and effort of the volunteers, and often on their expertise. With the help of the crowd, project initiators can pursue a goal that they would otherwise not be able to achieve.

### Ways of accessing the crowd

All crowd science projects are confronted with the challenge of accessing volunteers and motivating them to perform assigned tasks. Based on our case studies, we identified two main strategies for accessing the crowd: *crowd building* and *crowd harnessing*.

The *crowd building strategy* relies on recruiting volunteers around a specific question or issue. The idea is to build a community of volunteers who will perform specific tasks, reducing the project’s costs or scaling it up beyond what would otherwise be feasible. Project initiators reach volunteers through marketing campaigns via traditional or social media channels and, on a smaller scale, via outreach activities or word-of-mouth. The vast majority of the crowd science projects we analysed employ the crowd building strategy (see Table 2).

The *crowd harnessing strategy* relies on tapping into an already existing community. Among the projects we analysed, the ones that most closely approximated this strategy mobilised ornithologists (e.g. *Animal Tracker*) or scuba divers (e.g. *Tauchen für den Naturschutz*). The idea behind the strategy is to channel the power of an established community to achieve the project’s goals. This way of reaching volunteers requires the crowd science project to be set up around a specific topic and presupposes that the tasks will be designed in a way that is interesting for the community. The community harnessing strategy is employed by only a few of the projects we analysed (see Table 2), but it seems to be a strategy that can work well if there is a good match between the project’s objective and the interests of the community.

There are also hybrid forms that employ a mixture of crowd building and crowd harnessing strategies. For example, the *Animal Tracker* project builds on an existing community of ornithologists but needs to keep that community engaged and to recruit new members. Project initiators employing the crowd building strategy either need to find a way to keep volunteers interested in the project so that they keep coming back or, if they are counting on short-term volunteers, they need to find ways to maintain a steady flow of new volunteers. We present an overview of the strategies employed to access volunteers in the crowd science projects we analysed in our sample in Table 2.

An interesting aspect we identified with reference to accessing the crowd is an *effect-based approach*, whereby crowd science projects try to reach a goal by trial and error. The rationale of such approaches is that involving a large crowd of non-professionals will sooner or later produce comparable results to those that would be achieved by a small number of experts: ‘[W]e practically act via pure mass. So it can happen that quantity turns into quality’ (*ARTigo*). This means placing the focus on a large number of small contributions, rather than making efforts to keep a steady base of volunteers interested in the project in the hope of receiving regular contributions from more experienced volunteers. With this strategy, the sum of many one-time contributions can yield valuable results. A project initiator we interviewed used the following analogy to illustrate this idea:

I always have this comparison with a clay pigeon shooter […] who shoots down the things for you, but you have to pay him. Certainly you could do the same thing with 10,000 people, who are wildly shooting around; they would also hit the clay pigeons, even if only by chance. However, you could not do that in real life, because if 10,000 people stand on a field, they would end up shooting each other, but you can do that on the internet. That is the crazy thing. On the internet you can unleash huge crowds on something like that. (*ARTigo*)

The effect-based approach can best be employed in crowd science projects that are based on simple tasks and do not involve a learning curve. For example, the *ARTigo* project asks volunteers to add descriptive tags to images of paintings. In theory, it would be possible to solve this problem using a large number of volunteers who all participate only once. In this way, a large crowd of volunteers could potentially generate data that would surpass the contributions of a few dedicated volunteers in terms of quantity and possibly also in terms of overall quality. Moreover, data generated by a large crowd are more diverse than data generated by a core of ‘power volunteers’. The effect-based approach has the potential to break the typical pattern of a few very active people producing a substantial part of the outcome, while the majority of participants only produce a fraction of it. (‘And of course, things could be different, especially if we really had access to huge masses, then the potential would come true’ (*ARTigo*).) However, accessing volunteers willing to perform these tasks is a challenge, and therefore, this approach remains primarily a conceptual one. So far, *ARTigo* has employed a gamification approach, which ensures a steady flow of users who return to the game multiple times; however, this approach cannot be easily replicated by other platforms, and gamification approaches only hold true for projects that can break down tasks so they can constitute the building blocks of a game.

### Performing the tasks

Breaking down the tasks into manageable units is an essential element of crowd science. As our interviewee from the project *Datenerfassungssystem*, which is concerned with digitising historical sources, puts it, ‘Many people have told us: “I have found something here that interests me. I want to do something, too.” […] In this way the seeker who found the entry becomes a participant’.

In our analysis, we identified three broad types of tasks that project initiators allocate to volunteers: *annotating, collecting* and *producing*.

*Annotating* refers to adding a form of metadata to existing data, like tagging images or digitising text from historical sources. For example, volunteers participating in the *ARTigo* project tag photographs of paintings. In the project *Datenerfassungssystem*, volunteers transcribe historical lists of war casualties. These types of tasks are entirely Internet-based. In our case studies, tasks that involve annotating can be found in the following projects: *ARTigo, Datenerfassungssystem* and *Genealogisches Ortsverzeichnis* (*GOV*) (see Table 2).

*Collecting* means physically gathering data of some sort, such as catching a mosquito or taking a picture. This type of task typically applies to crowd science projects within the realm of the natural sciences. Volunteers are encouraged to go into the field and collect data and to submit the observations. The project *Mückenatlas*, for instance, asks volunteers to catch a mosquito without squashing it, to freeze it and subsequently to send it to the project coordinator. The other cases that involve collecting data are *Animal Tracker, Meilensteine der motorischen Entwicklung im Kleinkindalter* and *Tauchen für den Naturschutz* (see Table 2).

*Producing* involves creating new original content. In the project *Wissensdinge* for instance, this means writing a story about an object in the Natural History Museum in Berlin. Other examples are writing an article about an educational topic (e.g. *Bildungsexplosion*) or to translate ancient texts (e.g. *Open Philology*). This type of task is rare in the projects we analysed, and among crowd science initiatives generally, as it requires a considerable amount of commitment from the volunteers and is challenging to process on the part of the project initiators. This type of task signifies a form of crowd science in which volunteer contributions reach a certain threshold of originality that should qualify them for authorship of the kind typically offered by institutionalised science.

In addition to characterising the types of tasks, we have categorised them according to their degree of complexity. We distinguish between tasks that are *simple, medium* and *hard*. The majority of tasks that involve collecting or annotating can be classified as simple or medium. Breaking down a task into simple subtasks is a strategy that allows crowd science projects to distribute them to a large number of volunteers. Simple individual tasks allow projects to generate quality by means of quantity. Tasks that require volunteers to create new content tend to be complex and time-consuming, thus we classify them as hard. We present an overview of the various types of tasks and their degree of complexity in Table 3.

**Table 3.** Tasks.

Project	Tasks	Task category	Task complexity
Animal Tracker (AT)	Observing the behaviour of specific animals (e.g. stork, northern bald ibis) using GPS-data from the project’s app; uploading observations and pictures onto the database	Collecting	Medium
ARTigo – *Laien beschreiben Kunstwerke* (AG)	Tagging paintings as part of a game	Annotating	Simple
*Bildungsexplosion* (BE)	Writing articles about topics of their interest	Producing	Hard
DES – *Datenerfassungssystem* (DES)	Transcribing text from high resolution images of historical sources	Annotating	Simple/medium
GOV – *Genealogisches Ortsverzeichnis* (GOV)	Adding structured data (based on local knowledge or historical sources) into database	Annotating/collecting	Simple/medium
KLEKs (*Das Kulturlandschafts-Wiki*) (KW)	Adding new or editing existing elements (descriptions, pictures) referring to cultural landscapes in the database via an online editor	Annotating/collecting	Simple/medium
*Meilensteine der motorischen Entwicklung im Kleinkindalter* (ME)	Observing milestones of infant motor development	Collecting	Simple
*Mückenatlas* (MA)	Catching mosquitoes without damaging them, freezing them and mailing them to the project team	Collecting	Medium
*Open Philology* (OP)	Translating ancient Greek and Latin texts	Annotating/producing	Medium/hard
*Tauchen für den Naturschutz* (TN)	Mapping water quality in lakes while diving	Collecting	Medium/hard
*Verlust der Nacht – App* (VN)	Using app to measure night sky glow based on visibility of specific star constellations	Collecting	Simple
*WISSENSDINGE. Geschichten aus dem Naturkundemuseum Berlin* (WD)	Writing a story about an object from the Natural History Museum in Berlin	Producing	Hard

GPS: global positioning system.

Even though in most projects the tasks allocated to volunteers are rather simple, it is apparent from various statements in the interviews that project initiators have great respect for both the engagement and the expertise of the volunteers and highly value their contributions. As a project coordinator explained,

So there are numerous […] hobby ornithologists doing nothing else but driving around and watching birds. In terms of observations or general ornithological knowledge they are often even better than our scientists. (*Animal Tracker*)

### Quality assurance

Guaranteeing that the input received from volunteers is of high quality is another challenging aspect of crowd science. All projects in our sample take this issue into account, but only a few projects have developed efficient quality assurance strategies. Projects based on simple tasks involving collecting or annotating data can to some extent use automated quality control mechanisms. One such mechanism that was mentioned by several of the project initiators we interviewed was ‘double-keying’, that is, entering data (at least) twice in exactly the same form in order to be validated. Even though two people can easily repeat a common typo, in general, poor quality entries end up in the long tail of the data. As one of our interviewees explained,

The quality control is exclusively generated by validation through matching. Sometimes we get […] nonsense caused by misspellings, but it disappears somewhere at the end of the long tail. (*ARTigo*)

Projects that deal with more complex input rely on humans to ensure data quality. Even though volunteers can additionally be involved in a form of peer-review, none of the projects we examined had a working solution to fully automate the process of quality assurance for more complex tasks. This may conceivably change in the future as machine learning techniques become more sophisticated and easier to apply.

### Feedback mechanisms

Similar issues apply to feedback mechanisms. Feedback provision can be employed as a motivational tool that makes volunteers feel valued and encourages them to continue their engagement. In our case studies, most project initiators commented favourably on feedback mechanisms, but few projects seemed to have implemented a scalable feedback strategy. In most cases, feedback is channelled into conversational forms such as mailing lists or a discussion-forum where project coordinators and volunteers interact. Some project initiators make an extra effort by providing ‘power volunteers’ with personal feedback. One of our interviewees, for example, described how she sends personal thank-you letters to volunteers who provided her with samples. She remarked that these volunteers are interested in feedback that refers to the sample they sent rather than in a standard thank-you letter: ‘So each time I have to come up with something new to motivate them to continue. […] So you know, it’s like throwing them a little bit of bait’ (*Mückenatlas*).

While feedback mechanisms pose less of a problem for crowd science projects that employ a gamification strategy, other crowd science projects face the challenge of having to find ways to provide their volunteers with encouraging feedback. What seems important for the future is to develop scalable solutions by automating the feedback process while maintaining a personalised character.

## 5. Discussion

Crowd science, described here using a set of cases, is blurring the line between institutional science and civil society (Bonney et al., 2016; Riesch and Potter, 2014), but it is unlikely to fundamentally challenge institutional hierarchies. Growing political and economic demands on institutional science are manifesting themselves in increasing competition for resources, and there is great pressure to conduct research that has secondary social, political and economic benefits. Crowd science fits with this trend and it also confers social legitimacy on projects by involving non-experts. Crowd science can thus be understood as one manifestation of an on-going shift in the relationship between institutional science and society at large. Our findings on the *objectives* of crowd science projects suggest that both the revolutionary understanding of crowd science as a ‘bottom-up’ alternative to established institutional processes and as an evolutionary ‘top-down’ approach that integrates a crowd component into existing professional research workflows exist side by side. We found a high level of appreciation among institutional scientists for volunteers’ efforts. This suggests that the contributions of volunteers can be an asset for professional science. A closer interaction between professional and amateur scientists is considered attractive to career scientists because it may enable solutions to problems that would otherwise be intractable (Nature, 2015). It is also worth noting that the two groups do not need to share the same aims for a project to be successful. Models in which the outcome is effectively masked from the participants can thrive if the act of participating is entertaining or rewarding. Considering the momentum behind (mobile) online gaming and the large number of players that simple online games manage to attract, applying gamification techniques to crowd science projects could be a strategy to harness more of the potential of online technologies and also possibly a way to keep the crowd engaged beyond the purely educational aspects of such projects.

In analysing how crowd science projects *access volunteers*, we found that only a few of them mobilise existing communities. Motivating people to participate is one of the key challenges when setting up a crowd science project. It is therefore surprising that so many projects tackle this challenge by relying on their own power to draw a crowd. We propose that crowd science projects would benefit from more interaction with established communities. This, however, requires alignment and a high level of mutual understanding. Another approach that seems fruitful is to offer some form of reward to contributors (for instance, through feedback or authorship).

When looking at the *tasks* that crowd science projects ask their volunteers to perform, we found a predominance of relatively simple tasks. To counteract the argument that crowd science is just a form of cheap labour, projects could offer a broader array of tasks to be performed, including more complex ones. Such an approach would give motivated volunteers more options and serve as an example of how volunteers can play an integral role in research projects. At the same time, breaking down tasks into small – and consequently often simple units – constitutes the basis that allows for scaling up, automating quality assurance mechanisms as well as for applying gamification approaches. Striking a balance with regard to task complexity and the overall project set-up is an area for further explorations.

The formal system of scholarly communication represents a barrier that wards off outsiders. Professional science, as Dickel and Franzen (2016) point out, is highly selective and is only ‘open’ in a relative sense (p. 11). The problem of extending science to non-professionals (Collins and Evans, 2002) is complicated by a lowered barrier to scientific contribution via online platforms, but not in the sense that volunteers actively compete with professional scientists.

When studying the cases, we found that both *quality assurance* and *feedback mechanisms* were pivotal aspects for the success of crowd science projects. The literature on these topics is limited and only a few of the projects in our sample had a dedicated strategy to deal with these issues. For some projects, double-keying can be employed to perform quality assurance, but this method is not applicable to many settings. Here, more developed strategies are needed, as is research that evaluates these strategies and provides projects with guidelines. Feedback appears to be an essential part of crowd motivation. And while most project coordinators commented favourably on feedback in general, only a few have a scalable feedback strategy in place. This seems to be representative of the overall situation of crowd science. Crowd science is a relatively young phenomenon that is facing different challenges to traditional forms of citizen science. In many situations, project coordinators solve various problems ad hoc, without an initial strategic plan and without guidelines and best practice examples to refer to. Nevertheless, it is reasonable to assume that crowd science projects will professionalise over time. But in order for them to do so, more research on the topic is needed to provide project initiators with clear guidance.

And finally, it remains to be seen what impact current developments in crowd science will have on institutional science. Professional elites are resistant to change from the outside, and the unsolved problems in this area, such as ensuring the reliability of knowledge generated in crowd science platforms, may inhibit its success. The successful integration of crowd science into existing scientific practices, rather than the invention of alternative avenues of knowledge creation by those traditionally excluded, will be the yardstick by which the impact of crowd science will be measured.

## References

[bibr1-0963662516678514] Afuah A Tucci CL (2012) Crowdsourcing as a solution to distant search. Academy of Management Review 37(3): 355–375.

[bibr2-0963662516678514] Bayus BL (2013) Crowdsourcing new product ideas over time: An analysis of the Dell IdeaStorm community. Management Science 59(1): 226–244.

[bibr3-0963662516678514] Bonney R (1996) Citizen science: A lab tradition. Living Bird 15(4): 7–15.

[bibr4-0963662516678514] Bonney R Phillips TB Ballard HL Enck JW (2016) Can citizen science enhance public understanding of science? Public Understanding of Science 25(1): 2–16. 26445860 10.1177/0963662515607406

[bibr5-0963662516678514] Brossard D Lewenstein B Bonney R (2005) Scientific knowledge and attitude change: The impact of a citizen science project. International Journal of Science Education 27(9): 1099–1121.

[bibr6-0963662516678514] Castells M (2000) Materials for an exploratory theory of the network society1. British Journal of Sociology 51(1): 5–24.

[bibr7-0963662516678514] Collins HM (2014) Are We All Scientific Experts Now? (New human frontiers series). Cambridge: Polity.

[bibr8-0963662516678514] Collins HM Evans R (2002) The third wave of science studies: Studies of expertise and experience. Social Studies of Science 32(2): 235–296.

[bibr9-0963662516678514] Conrad CC Hilchey KG (2011) A review of citizen science and community-based environmental monitoring: Issues and opportunities. Environmental Monitoring and Assessment 176(1–4): 273–291. 20640506 10.1007/s10661-010-1582-5

[bibr10-0963662516678514] Cooper CB Dickinson J Phillips T Bonney R (2007) Citizen science as a tool for conservation in residential ecosystems. Ecology and Society 12(2): 11.

[bibr11-0963662516678514] Delaney DG Sperling CD Adams CS Leung B (2008) Marine invasive species: Validation of citizen science and implications for national monitoring networks. Biological Invasions 10(1): 117–128.

[bibr12-0963662516678514] Dickel S Franzen M (2016) The ‘Problem of Extension’ revisited: New modes of digital participation in science. Journal of Science Communication 15(1): 1–15.

[bibr13-0963662516678514] Dickinson JL Zuckerberg B Bonter DN (2010) Citizen science as an ecological research tool: Challenges and benefits. Annual Review of Ecology, Evolution, and Systematics 41(1): 149–172.

[bibr14-0963662516678514] Eisenhardt KM (1989) Building theories from case study research. Academy of Management Review 14(4): 532–550.

[bibr15-0963662516678514] Eisenhardt KM Graebner ME (2007) Theory building from cases: Opportunities and challenges. Academy of Management Journal 50(1): 25–32.

[bibr16-0963662516678514] Finke P (2014) Citizen Science: das unterschätzte Wissen der Laien [Citizen Science: das unterschätzte Wissen der Laien” Translation in eng: “Citizen Science: The underestimated knowledge of laypeople]. München: Oekom.

[bibr17-0963662516678514] Franzoni C Sauermann H (2014) Crowd science: The organization of scientific research in open collaborative projects. Research Policy 43(1): 1–20.

[bibr18-0963662516678514] Haklay M (2013) Citizen science and volunteered geographic information: Overview and typology of participation. In: Sui D Elwood S Goodchild M (eds) Crowdsourcing Geographic Knowledge: Volunteered Geographic Information (VGI) in Theory and Practice. Dordrecht: Springer, pp 105–122. Available at: 10.1007/978-94-007-4587-2_7

[bibr19-0963662516678514] Heer J Bostock M (2010) Crowdsourcing graphical perception: Using mechanical turk to assess visualization design. In: Proceedings of the SIGCHI conference on human factors in computing systems, Atlanta, GA, 10–15 April, pp. 203–212. New York, NY: ACM.

[bibr20-0963662516678514] Hochachka WM Fink D Hutchinson RA Sheldon D Wong W-K Kelling S (2012) Data-intensive science applied to broad-scale citizen science. Trends in Ecology & Evolution 27(2): 130–137. 22192976 10.1016/j.tree.2011.11.006

[bibr21-0963662516678514] Howe J (2006) The rise of crowdsourcing. Wired Magazine 14(6): 1–4. Available at: https://www.wired.com/2006/06/crowds/

[bibr22-0963662516678514] Irwin A (1995) Citizen Science: A Study of People, Expertise, and Sustainable Development (Environment and society). London; New York, NY: Routledge.

[bibr23-0963662516678514] Jick TD (1979) Mixing qualitative and quantitative methods: Triangulation in action. Administrative Science Quarterly 24(4): 602–611.

[bibr24-0963662516678514] Land-Zandstra AM Devilee JLA Snik F Buurmeijer F van den Broek JM (2016) Citizen science on a smartphone: Participants motivations and learning. Public Understanding of Science 25(1): 45–60. 26346340 10.1177/0963662515602406

[bibr25-0963662516678514] Leimeister JM Huber M Bretschneider U Krcmar H (2009) Leveraging crowdsourcing: Activation-supporting components for IT-based ideas competition. Journal of Management Information Systems 26(1): 197–224.

[bibr26-0963662516678514] Merriam-Webster (2016) Crowdsourcing | Definition of crowdsourcing by Merriam-Webster. Available at: http://www.merriam-webster.com/dictionary/crowdsourcing

[bibr27-0963662516678514] Meyer ET Schroeder R (2015) Knowledge Machines: Digital Transformations of the Sciences and Humanities (Infrastructures). Cambridge, MA: The MIT Press.

[bibr28-0963662516678514] Miles MB Huberman AM (1984) Qualitative Data Analysis: A Sourcebook of New Methods. London: SAGE.

[bibr29-0963662516678514] Mirowski P (2011) Science-Mart: Privatizing American Science. Cambridge, MA: Harvard University Press.

[bibr30-0963662516678514] Nature E (2015) Rise of the citizen scientist. Nature 524(7565): 265. 10.1038/524265a 26289171

[bibr31-0963662516678514] Nielsen MA (2012) Reinventing Discovery: The New Era of Networked Science. Princeton, NJ: Princeton University Press Available at: http://public.eblib.com/choice/publicfullrecord.aspx?p=773462 (accessed 23 September 2016).

[bibr32-0963662516678514] Poetz MK Schreier M (2012) The value of crowdsourcing: Can users really compete with professionals in generating new product ideas? Journal of Product Innovation Management 29(2): 245–256.

[bibr33-0963662516678514] Raddick MJ Bracey G Gay PL Lintott CJ Murray P Schawinski K (2010) Galaxy Zoo: Exploring the motivations of citizen science Volunteers. Astronomy Education Review 9(1). Available at: http://www.portico.org/Portico/article?article=pgg3ztfdp8z (accessed 23 September 2016).

[bibr34-0963662516678514] Reed MS Kenter J Bonn A Broad K Burt TP Fazey IR (2013) Participatory scenario development for environmental management: A methodological framework illustrated with experience from the UK uplands. Journal of Environmental Management 128: 345–362. 23774752 10.1016/j.jenvman.2013.05.016

[bibr35-0963662516678514] Riesch H Potter C (2014) Citizen science as seen by scientists: Methodological, epistemological and ethical dimensions. Public Understanding of Science 23(1): 107–120. 23982281 10.1177/0963662513497324

[bibr36-0963662516678514] Sauermann H Franzoni C (2015) Crowd science user contribution patterns and their implications. Proceedings of the National Academy of Sciences 112(3): 679–684. 10.1073/pnas.1408907112 PMC4311847 25561529

[bibr37-0963662516678514] Silvertown J (2009) A new dawn for citizen science. Trends in Ecology & Evolution 24(9): 467–471. 19586682 10.1016/j.tree.2009.03.017

[bibr38-0963662516678514] Stehr N (2002) Knowledge and Economic Conduct: The Social Foundations of the Modern Economy (Studies in comparative political economy and public policy). Toronto, ON, Canada: University of Toronto Press.

[bibr39-0963662516678514] Sullivan BL Wood CL Iliff MJ Bonney RE Fink D Kelling S (2009) eBird: A citizen-based bird observation network in the biological sciences. Biological Conservation 142(10): 2282–2292.

[bibr40-0963662516678514] Tulloch AIT Possingham HP Joseph LN Szabo J Martin TG (2013) Realising the full potential of citizen science monitoring programs. Biological Conservation 165: 128–138.

[bibr41-0963662516678514] Wechsler D (2014) Crowdsourcing as a method of transdisciplinary research – Tapping the full potential of participants. Futures 60: 14–22.

[bibr42-0963662516678514] Yin RK (2013) Case Study Research: Design and Methods. London: SAGE.

[bibr43-0963662516678514] Zook M Graham M Shelton T Gorman S (2010) Volunteered geographic information and crowdsourcing disaster relief: A case study of the Haitian earthquake. World Medical & Health Policy 2(2): 6–32.

